# Supplementary material for: An amplicon panel for high-throughput and low-cost genotyping of Pacific oyster
Source: G3 (Bethesda). 2024 Jun 13;14(9):jkae125. doi: 10.1093/g3journal/jkae125 (PMC11373646; doi:10.1093/g3journal/jkae125)
Supplement: jkae125_Supplementary_Data [file jkae125_supplementary_data.zip › Supplemental_Material_Legends_G3-2024-404881.docx]

**Supplemental Material**

File S1. Amplicon panel design file including target variant with the reference and alternate allele and flanking genomic sequence as well as genomic coordinates.

File S2. Marker names from ddRAD-seq study, reason for selection in panel, and panel marker name.

File S3. Target SNP file with Cgig_v.1.0 target sites used for variant calling.

File S4. Target design windows that aligned more than once to the reference genome with bowtie2 or bwa mem.

File S5. Per locus *H*_OBS_ and *F*_ST_ from the amplicon panel pilot study.

File S6. Per locus Hardy-Weinberg equilibrium statistics for wild or naturalized populations.

File S7. Number of loci genotyped per individual in the population genetic dataset before filters, and in each parentage dataset (VIU OFR5 and MBP CHR8).

File S8. Parent-offspring relationships for the VIU OFR5 and MBP CHR8 families.

File S9. Per cross identities of null alleles and overlaps between the crosses.

Supplemental Figures and Tables. Figure S1-S8 and Table S1-S3.
